# Angular Distribution of Ion Products in the Double Photoionization of Propylene Oxide

**DOI:** 10.3389/fchem.2019.00621

**Published:** 2019-09-11

**Authors:** Stefano Falcinelli, Marzio Rosi, Fernando Pirani, Davide Bassi, Michele Alagia, Luca Schio, Robert Richter, Stefano Stranges, Nadia Balucani, Vincent Lorent, Franco Vecchiocattivi

**Affiliations:** ^1^Department of Civil and Environmental Engineering, University of Perugia, Perugia, Italy; ^2^Department of Chemistry, Biology and Biotechnologies, University of Perugia, Perugia, Italy; ^3^Department of Physics, University of Trento, Trento, Italy; ^4^IOM-CNR Tasc, Trieste, Italy; ^5^Department of Basic and Applied Sciences for Engineering (SBAI), University of Rome “Sapienza”, Rome, Italy; ^6^Elettra-Sincrotrone Trieste, Trieste, Italy; ^7^Department of Chemistry and Drug Technologies, University of Rome “Sapienza”, Rome, Italy; ^8^Laboratoire de physique des lasers, Université Paris 13 (UP13) - Institut Galilée - CNRS LPL UMR7538, Villetaneuse, France

**Keywords:** double photoionization, molecular dications, coincidence technique, propylene oxide, chiral, synchrotron radiation, angular distribution, astrochemistry

## Abstract

A photoelectron-photoion-photoion coincidence technique, using an ion imaging detector and tunable synchrotron radiation in the 18.0–37.0 eV photon energy range, inducing the ejection of molecular valence electrons, has been applied to study the double ionization of the propylene oxide, a simple prototype chiral molecule. The experiment performed at the Elettra Synchrotron Facility (Trieste, Italy) allowed to determine angular distributions for ions produced by the two-body dissociation reactions following the Coulomb explosion of the intermediate (C_3_H_6_O)^2+^ molecular dication. The analysis of the coincidence spectra recorded at different photon energies was done in order to determine the dependence of the **β** anisotropy parameter on the photon energy for the investigated two-body fragmentation channels. In particular, the reaction leading to ${\text{CH}}_{3}^{+}$ + C_2_H_3_O^+^ appears to be characterized by an increase of **β**, from **β** ≈ 0.00 up to **β** = 0.59, as the photon energy increases from 29.7 to 37.0 eV, respectively. This new observation confirms that the dissociation channel producing ${\text{CH}}_{3}^{+}$ and C_2_H_3_O^+^ final ions can occur with two different microscopic mechanisms as already indicated by the bimodality obtained in the kinetic energy released (KER) distributions as a function of the photon energy in a recent study. Energetic considerations suggest that experimental data are compatible with the formation of two different stable isomers of C_2_H_3_O^+^: acetyl and oxiranyl cations. These new experimental data are inherently relevant and are mandatory information for further experimental and theoretical investigations involving oriented chiral molecules and linearly or circularly polarized radiation. This work is in progress in our laboratory.

## Introduction

In life science a basic role is played by the left-right dissymmetry, at a macroscopic as well as at the microscopic level. In terrestrial life the unknown origin of homochirality and of the enantio-selectivity in biochemical processes involving chiral molecules are among the most intriguing aspects in natural phenomena (Engel and Macko, 1997; Pizzarello and Groy, 2011). Therefore, a big role is played by the investigation of molecular enantiomeric nature on various fields of chemistry, as for example the heterogeneous enantioselective catalysis, the photochemical asymmetric synthesis, the study of drug activity and enzymatic catalysis, the chiral surface science involving supramolecular assemblies.

Nowadays, and already for several decades, a wide number of experiments are carried out world-wide using circularly polarized tunable light sources of high intensity as synchrotron radiation and, in order to detect chirality in molecules, imaging photoelectron circular dichroism (Turchini et al., 2004; Stranges et al., 2005; Piancastelli et al., 2007; Alberti et al., 2008; Janssen and Powis, 2014). Moreover, techniques of molecular alignment with the pioneering work done by Zare et al. (Sinha et al., 1974; Caldwell and Zare, 1977) and by B. Friedrich and D. R. Herschbach (Friedrich and Herschbach, 1991; Friedrich et al., 1991) have been found of great relevance to control the stereodynamics of elementary chemical processes occurring in the gas phase and at surfaces, and are arguably crucial in chirality issues (Falcinelli et al., 2018a). In particular, previous studies (Caldwell and Zare, 1977; Karny et al., 1978; Sanders and Anderson, 1984; Pullman et al., 1990; Aquilanti et al., 1994) indicate that molecular directionality and alignment should strongly amplify dichroism and provide stereodynamical mechanisms for discrimination of enantiomers, other than via circularly polarized light (Herwig et al., 2013; Pitzer et al., 2013; Tia et al., 2017). Very recently, in our laboratory we are working, to couple a mechanical molecular velocity selector, specifically constructed *ad hoc* (able to control the velocity dependence of the molecular alignment) with a PEPIPICO (photo-electron-photo-ion-photo-ion coincidence) imaging device, in order to be used with a synchrotron radiation (Falcinelli et al., 2018a), delivering tunable intense photon sources with high degree of both linearly and circularly polarized light of both helicities. Double photoionization studies on propylene oxide enantiomeric molecules have been done by exploiting Auger spectroscopy (Piancastelli et al., 2007; Alberti et al., 2008). In these experiments, using high photon energy synchrotron radiation (in the 289–380 eV energy range), the authors indirectly estimated the first double ionization threshold, allowing the observation of a weak circular dichroism effect (Alberti et al., 2008). In our recent experimental work performed at the Synchrotron Radiation Facility of Elettra (Trieste, Italy) we were able to identify six two body fragmentation channels produced by double photoionization of propylene oxide in the 18.0–37.0 eV photon energy range, including their relative threshold energies (Falcinelli et al., 2018a). Such two body fragmentation channels involve the direct ejection of two valence electrons, and allowed us to determine with more accuracy the first double photoionization energy of propylene oxide, obtaining the values of 28.3 ± 0.1 which is 0.9 eV below the previous indirect estimation of 29.2 eV by Auger spectroscopy (Piancastelli et al., 2007; Alberti et al., 2008).

Recently, using the photoelectron circular dichroism (PECD) as an established technique for chiral differentiation, interesting PECD data have been recorded in single photoionization experiments on enantiomerically pure trifluoromethyl-oxirane and methyl-oxirane (Garcia et al., 2014).

In 2016 propylene oxide has been discovered as the first chiral molecule in space by McGuire et al. by means of astronomical detection in absorption toward the Galactic center (McGuire et al., 2016). This important result highlights the relevance of a deep and full investigation of the possible fragmentation processes following the microscopic fragmentation dynamics by Coulomb explosion of propylene oxide (C_3_H_6_O)^2+^ molecular dications produced by VUV double photoionization.

After the pioneering work of Hipple concerning the spontaneous dissociation processes of ions (Hipple, 1948), the importance of the fragmentation dynamics following single and double photodissociation photoionization processes has been investigated in a number of papers, respectively by R. N. Zare, who first fully described the dynamics of molecular photodissociation (Zare and Herschbach, 1962, 1963; Zare, 1972; Greene and Zare, 1982), and by J. H. D. Eland et al. (Eland and Treves-Brown, 1992; Field and Eland, 1993; Eland, 2006). Following such basic contributions, various authors contributed to this Research Topic (Fišer et al., 2009; Price et al., 2017), also highlighting the role of singly and doubly charged ions in the chemistry of upper planetary atmospheres (Thissen et al., 2011; Falcinelli et al., 2014, 2016a) and in space (Ascenzi et al., 2004; Bartolomei et al., 2008; Skouteris et al., 2015). Furthermore, dissociation dynamics of multiply charged ions has been studied [see for example Geronés et al. and references therein (Geronés et al., 2007)] with related implications on chemistry of the interstellar medium (Geronés et al., 2012). In particular, molecular metastable dications have attracted the attention of the scientific community after the pioneering valence bond calculations on ${\text{He}}_{2}^{2+}$ carried out by Linus Pauling in 1933 (Pauling, 1933), since the possibility to be used as species able to store energy at a molecular level (Nicolaides, 1989; Boldyrev and Simons, 1992; Falcinelli et al., 1996; Tosi et al., 1999). These species can in principle be used as a new kind of alternative propellants since they are able to release a considerable amount of energy (up to about 10 eV) following their unimolecular fragmentation process by Coulomb explosion. In fact, this released amount of energy is exceptionally high being much greater than the energy obtainable from other important gas-phase reactions such as the one involving H_2_ and O_2_ producing water which is exothermic by ~2.52 eV (Nicolaides, 1989).

In this paper, a double photoionization study of propylene oxide—a prototype chiral molecule—is presented. The results of this experiment provide a solid basis to plan more ambitious future studies capable to highlight possible differences characterizing the interaction of polarized light with chiral systems, such as the angular distribution of both photo-emitted electrons and produced ions. In order to achieve such a goal, first of all, we have recently started by the use of linearly polarized synchrotron radiation, such as the one available at the Circular Polarization (CiPo) beamline at the ELETTRA Synchrotron Facility of Trieste (Italy). We performed double photoionization experiments involving a racemic mixture of propylene oxide, using the same ARPES (Angle-Resolved PhotoEmission Spectroscopy) apparatus (see next section) successfully employed in previous studies at the Elettra “GasPhase” beamline, since almost two decades (Alagia et al., 2002, 2004a,b,c, 2011a,b, 2013a; Falcinelli et al., 2017a,b). Even if the use of a racemic mixture of propylene oxide could appear as a limitation in our experiment, the recorded data here presented are very important for further experimental investigations involving single enantiomers that are planned in next future. In this preliminary work we were able to identify the most abundant two-body fragmentation channels produced by the photodissociation process of the neutral molecular C_3_H_6_O precursor. In such an experiment the use of a single photon with an energy content around the threshold of the double photoionization of the molecule under study is able to extract a pair of valence electrons. This allows the formation of an intermediate molecular dication able to dissociate by Coulomb explosion, as we have discussed in our previous investigations (Alagia et al., 2004a, 2006a, 2007, 2010; Falcinelli et al., 2014, 2016a,b), even if a debate is still open on whether the multiply charged molecule obeys pure Coulomb explosion model during its breakup or not (Sheehy and DiMauro, 1996; Mathur, 2004). Preliminary data already published on the double photoionization of propylene oxide concern: (i) the threshold energy for the recorded six two-body dissociation channels producing C_2_ ${\text{H}}_{4}^{+}$/CH_2_O^+^, C_2_${\text{H}}_{3}^{+}$/CH_3_O^+^, ${\text{CH}}_{2}^{+}$/C_2_H_4_O^+^, ${\text{CH}}_{3}^{+}$/C_2_H_3_O^+^, O^+^/C_3_${\text{H}}_{6}^{+}$, and OH^+^/C_3_${\text{H}}_{5}^{+}$ ion pairs; (ii) their relative cross sections as a function of the investigated photon energy; and (iii) the kinetic energy released (KER) distributions of each ion pair products as a function of the photon energy (Falcinelli et al., 2018a). In the present paper we report on the angular distributions of ion pair products formed in the two-body fragmentation reactions mentioned above. The measured angular distributions as a function of the photon energy (in the range of 18.0–37.0 eV) and the determination of the related anisotropy parameter **β** allowed us to confirm the existence of two possible microscopic mechanisms for the fragmentation channel producing ${\text{CH}}_{3}^{+}$/C_2_H_3_O^+^ ion pairs, as highlighted by the bimodality behavior in the recorded KER distributions already published (Falcinelli et al., 2018a) and by recent energetic considerations based on the high level coupled-cluster single and double plus perturbative triples CCSD(T) preliminary calculations (Falcinelli et al., 2019b).

## Experimental

Experiments have been carried out at the ELETTRA Laboratory, using the ARPES end station at the CiPo beamline. For a detailed description of the beamline and the end station the reader can refer to Derossi et al. (1995) and Alagia et al. (2006a, 2013b), respectively. Main characteristics of the used PEPIPICO time-of-flight technique have been described in previous studies producing and characterizing dications in the double photoionization of various neutral precursor molecules (Alagia et al., 2006b, 2009; Falcinelli et al., 2016b). Here, only a brief summary is given of the used experimental apparatus, and related procedures.

The operating pressure of the ARPES end station is 10^−7^-10^−8^ mbar. The high intensity energy tunable synchrotron light beam crosses at right angle an effusive molecular beam of propylene oxide. Product ions and photoelectrons produced by each double photoionization event are detected in coincidence. The experiment used a synchrotron light scanned in the 18.0–37.0 eV investigated photon energy range at the “CiPo” beamline with the light polarization vector which is in the synchrotron ring plane and perpendicular to the used time-of-flight (TOF) mass spectrometer. The Normal Incidence Monochromator (NIM), equipped with two different (2,400 l/mm gold and 1,200 l/mm aluminum coated) holographic gratings, allows to cover the 8.0–37.0 eV energy range. The resolution over this range is about 2.0–1.5 meV. The use of the NIM geometry was adopted to reduce spurious effects, due to ionization by photons from higher orders of diffraction. The adoption of the NIM at the “CiPo” beamline, together with the emission spectrum of the electromagnetic wiggler allows to probe a lower photon energy range with respect to the linearly polarized light available at the “GasPhase” beamline. Despite that the “CiPo” beamline can provide both linearly and circularly polarized radiation, since the present experiment involved a racemic mixture of propylene oxide precursor molecules, we recorded the angular distributions of produced ion pairs employing the linearly polarized component. The use of circularly polarized light could be relevant in future experiments when we will try to record possible differences in the angular and energy distribution of fragment ions at different photon energies, employing the two different enantiomers of propylene oxide.

In the experiment we monitored (i) the incident photon flux; (ii) the gas pressure, and (iii) the ion yields for each investigated channel, which are divided for the total ion yield when we are interested to obtain the branching ratios as a function of the photon energy (Falcinelli et al., 2018a). The effusive molecular beam is produced by a 1.0 mm stainless steel needle nozzle fed by a racemic mixture−99% nominal purity—of commercial propylene oxide. At 20°C propylene oxide is liquid, and its vapor pressure is 0.59 bar. A needle valve is placed between the glass cylinder containing the propylene oxide and the nozzle to optimize the molecular beam source pressure.

The electron-ion-ion coincidence technique was used to detect photoions in coincidence with photoelectrons, ejected from the photoionized neutral molecular precursor. The ion extraction and detection system, as well as the data analysis procedure, have been described in detail elsewhere (Lavollée, 1999; Alagia et al., 2012a). Such a device was especially designed to properly measure the cation photofragment momentum vectors in many body dissociation processes (Lavollée, 1999), and consists of a TOF spectrometer equipped with a position sensitive detector which is composed of a stack of three impedance matched micro-channel-plates (MCP) with a multi-anode array arranged in 32 rows and 32 columns.

**Figure 1:**
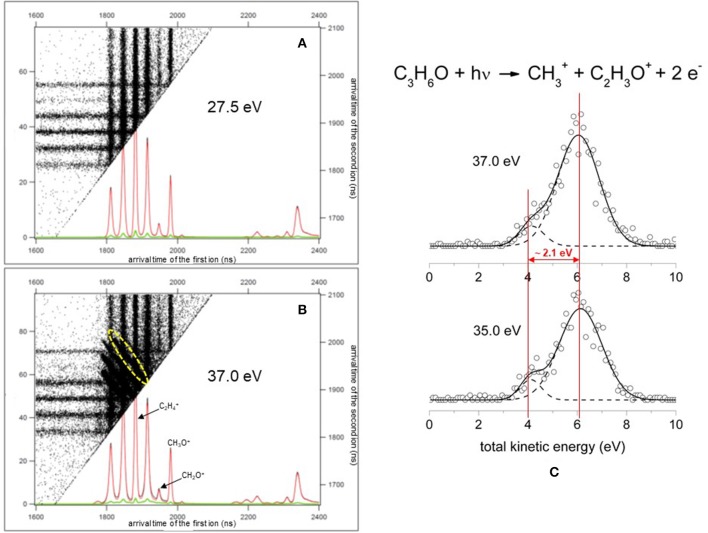
A portion of typical coincidence plots obtained in the photoelectron-photoion-photoion coincidence experiment. **(A)** Is related to a spectrum recorded at a photon energy of 27.5 eV, below the double ionization threshold energy (28.3 eV), where is only present the signal due to single ionization events together with some background. **(B)** Is a spectrum recorded at a photon energy of 37.0 eV where the signal of the double coincidences is well-evident (the diagonal traces in the figure—see text); the yellow dashed oval points out the region of the coincidence spectrum used for the evaluation of both KER and angular distributions in the case of the two fragmentation channels leading to C_2_${\text{H}}_{3}^{+}$/CH_3_O^+^ and C_2_${\text{H}}_{4}^{+}$/CH_2_O^+^ ion pairs. **(C)** A portion of total KER distribution for the production of ${\text{CH}}_{3}^{+}$/C_2_H_3_O^+^ ion pair products formed in the double dissociative photoionization of propylene oxide at a photon energy of 35.0 and 37.0 eV. The best fit (full line) of experimental data (open circles) is obtained using the sum of two different Gaussian functions (dashed lines) highlighting a bimodality generated by the presence of two different reaction mechanisms (Falcinelli et al., 2018a).

In general, our experiments concern the double ionization of simple molecules using a photon energy around the ionization threshold. In such conditions we are able to detect and analyse with a momentum matching procedure (see below) only the two-body fragmentation channels following the Coulomb explosion of the intermediate molecular dication. In particular, by a careful analysis of the coincidence plots recorded for each investigated photon energy we are able to obtain both KER and angular distribution maps of the product ions coming from the Coulomb explosion of the intermediate molecular dication formed by the double photoionization of the neutral propylene oxide precursor. Exploiting a procedure, suggested by Lundqvist et al. (1995) and by Field and Eland (1993), the kinetic energy of the final products, released into the two photofragment ions, as well as the lifetime of the intermediate dication can be extracted, as we have done in the case of the Coulomb explosion of (N_2_O)^2+^ and (CO_2_)^2+^ dications following the double photoionization of nitrous oxide (Alagia et al., 2006a) and carbon dioxide (Alagia et al., 2009) molecules, respectively. In particular, this goal can be obtained exploiting a momentum matching analysis on the peak for each ion pair in the coincidence spectra measured at all the investigated photon energies (Falcinelli et al., 2018a). The adopted computational procedure used a momentum matching filter to discriminate true coincidences related to each recorded fragmentation channels, produced by Coulomb explosion of the intermediate propylene oxide (C_3_H_6_O)^2+^ dication. Typical coincidence plots obtained in our PEPIPICO experiment are shown in Figure 1A is related to an accumulation run performed at a photon energy of 27.5 eV, which is below the double ionization threshold energy (28.3 eV) of propylene oxide, where only the signal due to single ionization events can be observed (Figure 1B) is an ion-ion coincidence plot recorded at a photon energy of 37.0 eV where the signals of the double coincidences are well-evident as the diagonal traces centered at the crossing of the two different arrival times of fragment product ions to the ion position MCP detector. Figure 1B is only a portion of the full recorded coincidence spectrum. In this plot the diagonal traces are related to the double coincidences signal of the two-body fragmentation channels. In particular, the yellow dashed oval in Figure 1B points out the region used for the evaluation of both KER and angular distributions in the case of the two fragmentation channels leading to C_2_${\text{H}}_{3}^{+}$/CH_3_O^+^ and C_2_${\text{H}}_{4}^{+}$/CH_2_O^+^ ion pairs. In the plot the double coincidences are reported as a function of the two arrival times (indicated in ns) of ions to the ion imaging detector which are produced from the same double photoionization event followed by Coulomb explosion of the intermediate (C_3_H_6_O)^2+^ dication. Such dots are extracted and discriminated from either background and single ionization signals in order to be used in the momentum matching filter analysis isolating only pulses due to true double coincidences. To achieve this goal the succeeding analytical condition has been employed:

(1) s≤px1,22+py1,22+pz1,22|p1|+|p2|

Where ***p***_***x*1, 2**_, ***p***_***y*1, 2**_, and ***p***_***z*1, 2**_ stand for the sum of projections of p1 and p2 momentum vectors of the final **1** and **2** ion pairs produced by the two-body fragmentation processes following the double photoionization of the neutral precursor molecule. In the analytical procedure, in which TOF and position on the detector of the arrival ion allow us to extract, without ambiguity, complete information concerning the linear momentum (***p***_***x***_, ***p***_***y***_, ***p***_***z***_) for each product ion, and consequently its angular and energy distribution, we used a value of S ≤ 0.1 in order to fully subtract the false coincidences. Such a condition (S ≤ 0.1) allowed us to subtract the background signal in the relative cross section for each recorded two-body fragmentation channel. In particular, S = 0.1 is an empirical factor which is the result of a compromise between a good statistics of the recorded signal and the background subtraction. Following such a procedure we were able to extract the KER distributions of any pair of fragment ions formed in two-body dissociation processes generated by double photoionization of any neutral precursors. For such a purpose we used the well-tested method suggested by Lundqvist et al. (1995) already successfully applied in analogous experiments involving different systems (Alagia et al., 2011a,b, 2012a,b; Falcinelli et al., 2016b). As an example, Figure 1C reports a portion of total KER distribution (with an energy resolution of ~ 0.2 eV) for the production of ${\text{CH}}_{3}^{+}$/C_2_${\text{H}}_{3}^{+}$ ion pair products formed in the double dissociative photoionization of propylene oxide at a photon energy of 35.0 and 37.0 eV. To have an overview of recorded KER distributions for all investigated fragmentation channels produced in the double-photoionization experiments of propylene oxide molecules as a function of the photon energy in the 18.0–37.0 eV range the reader can refer to a recently published paper (Falcinelli et al., 2018b).

## Results and Discussion

Previous experiments performed at the ELETTRA Synchrotron Facility, allowed us to perform a direct measurement of the first double photoionization energy for propylene oxide: 28.3 ± 0.1 eV (Falcinelli et al., 2018a). Six different two-body fragmentation channels were recorded in our mass spectra in the investigated photon energy range (18–37 eV) with their respective relative abundances and threshold energies, as it follows:

$$\begin{array}{l} C_{3}H_{6}O+hv \end{array}$$

(2) $$\begin{array}{l} \to C_{2}H_{4}^{+}+CH_{2}O^{+}+2e^{-}\;66.70\%\;h\nu =28.3\pm 0.1eV \end{array}$$

(3) $$\begin{array}{l} \to CH_{2}^{+}+C_{2}H_{4}O^{+}+2e^{-}\;7.84\%\;h\nu =28.5\pm 0.1eV \end{array}$$

(4) $$\begin{array}{l} \to CH_{3}^{+}+C_{2}H_{3}O^{+}+2e^{-}\;5.00\%\;h\nu =29.0\pm 0.3eV \end{array}$$

(5) $$\begin{array}{l} \to O^{+}+C_{3}H_{6}^{+}+2e^{-}\;1.59\%\;h\nu =29.0\pm 0.2eV \end{array}$$

(6) $$\begin{array}{l} \to C_{2}H_{3}^{+}+CH_{3}O^{+}+2e^{-}\;18.70\%\;h\nu =29.2\pm 0.1eV \end{array}$$

(7) $$\begin{array}{l} \to OH^{+}+C_{3}H_{5}^{+}+2e^{-}\;0.17\%\;h\nu =32.1\pm 0.3eV \end{array}$$

The highest abundance of the Reaction (2) is due to the direct breaking of the two C−O and C−C bonds in the central C atom of the intermediate (C_3_H_6_O)^2+^ molecular dication. As already discussed in detail in a previous paper where relative cross sections as a function of the photon energy are reported and discussed for all Reactions (2)–(7) (Falcinelli et al., 2018a), this main important Reaction (2) results in the formation of the most abundant C_2_${\text{H}}_{4}^{+}$ produced ion in single ionization experiments on propylene oxide, together with CH_2_O^+^ being identified in previous experiments by others authors (Gallegos and Kiser, 1961; Liu et al., 1999). On the other hand, the two-body dissociation Reactions (6) and (7) are characterized by a concerted fragmentation with a hydrogen migration. Such a microscopic mechanism seems to be difficult to achieve in the case of the hydrogen shift toward the oxygen atom [Reaction (7)], as indicated by the low percentage formation of about 0.17%, but it appears of considerable amount for Reaction (6) which involves hydrogen migration toward the carbon atom, being the second more probable recorded fragmentation reaction with its 18.7%. In particular, in the case of the Reaction (6) in order to confirm this hypothesis it would be very useful to carry out an experiment with selectively deuterated propylene oxide (Otley et al., 2011). Future investigations in this direction are planned by our research group at the ELETTRA Synchrotron Facility.

It has to be noted that the ${\text{CH}}_{3}^{+}$ formation by Reaction (4) could involve two different microscopic mechanisms as already mentioned in a recent paper (Falcinelli et al., 2018a), where the reported KER distributions of ${\text{CH}}_{3}^{+}$ + C_2_H_3_O^+^ final ions clearly show a bimodal behavior. In that paper we anticipated that further experiments were needed in order to clarify the two different microscopic paths involved. With this aim we have performed the present experiment based on the measure of the angular distributions of all ion pair products produced by Reactions (2)–(7) with respect the polarization vector of the used synchrotron radiation. For this purpose we have used the PEPIPICO technique and the related data analysis procedure described in the previous section has been widely employed by our research group during the last 15 years studying the dissociative double photoionization of different simple molecules, as: (i) N_2_O, where we found a very strong anisotropy distribution for both ion pair products of the two dissociative channels leading to N^+^+ NO^+^ and O^+^ + ${\text{N}}_{2}^{+}$ indicating that N_2_O molecules ionize when their axis is parallel to the light polarization vector, and the fragment ions are separating in a time shorter than the dication rotational period of about 10^−11^ s (Alagia et al., 2007); (ii) CO_2_ with the production of intermediates ${\text{CO}}_{2}^{2+}$ dications having a lifetime of ~3.1 μs which form O^+^ ions with a very high kinetic energy content able to explain their escape from the upper atmosphere of Mars (Alagia et al., 2010; Falcinelli et al., 2016a); and (iii) C_2_H_2_ where a quite evident change in the anisotropy **β** parameter (see below) related to the symmetric CH^+^+ CH^+^ fragmentation reaction indicating that two microscopic mechanisms involving several electronic states of the intermediate C_2_${\text{H}}_{2}^{2+}$ molecular dication are operative (Alagia et al., 2012b). Recently others research groups using the same PEPIPICO technique performed interesting experiments studying the photodissociation of simple organic molecules, as alcohols (Bava et al., 2015), carboxylic acid (Arruda et al., 2016), and perfluoropropionic acid (Martínez et al., 2018).

Using our ions position sensitive detector we obtained two dimensional (2D) images which are related to the photoelectron-ion-ion coincidence dots on the recorded coincidence plots at each investigated photon energy as those reported in Figures 1A,B. As already mentioned above, such dots are generated from pulses of dissociation products recorded as a function of their arrival time and position on the MCP detector plane. The recorded 2D images display the projection of the 3D distributed ion products on the plane of the MCP, and their best fit simulation procedure allowed us to determine the so called anisotropy parameter **β** according to the following equation (Zare, 1972; Dehmer and Dill, 1978; Ashfold et al., 2006):

(8) $$I\left(\theta \right)=\frac{\sigma_{tot}}{4\pi }\left[1+\frac{\beta }{2}\;\left(3\cos^{2}\theta -1\right)\right]$$

**Figure 2:**
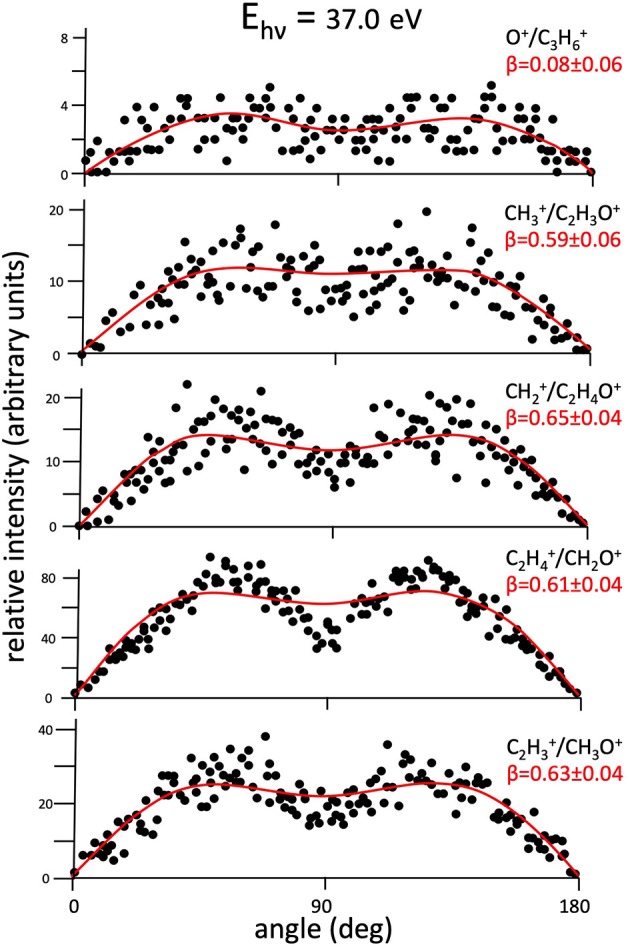
Angular distributions of ion pair products formed by main recorded two-body fragmentation reactions [Reactions (2)–(6)] following the double photoionization of propylene oxide at a photon energy of 37.0 eV. Dots intensity in the ordinate axis are in arbitrary units, and the error bars are omitted for clarity being of the same order of magnitude of the dot dimensions. In each panel is also reported the best fit calculation (full line) on the basis of Equation (8) giving the related anisotropy parameter **β** (see text).

**Figure 3:**
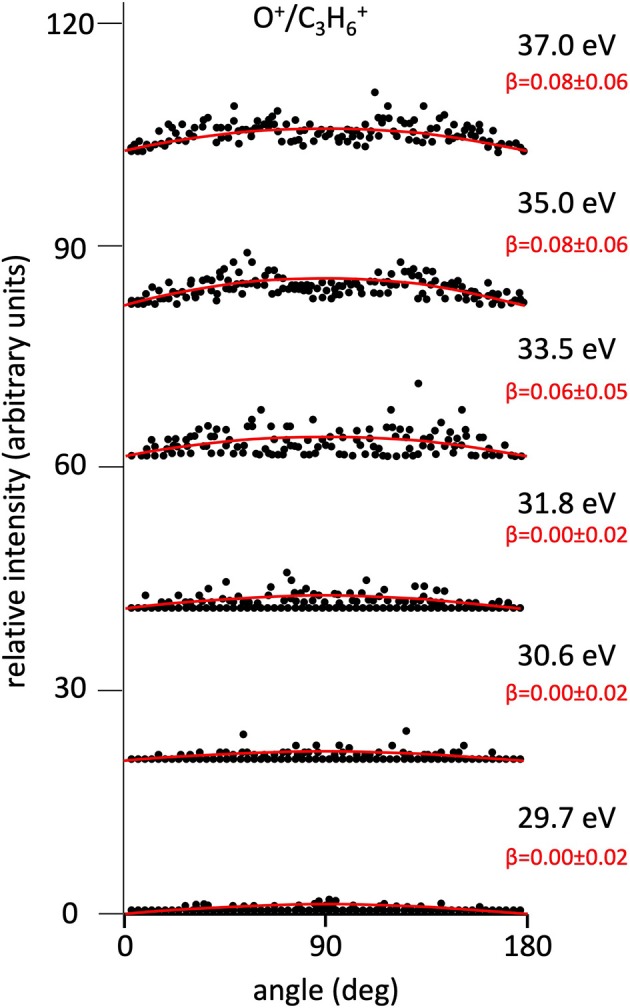
Angular distributions of O^+^/C_3_${\text{H}}_{6}^{+}$ ion pair products formed by Reaction (5) as a function of the photon energy. Dots intensity in the ordinate axis are in arbitrary units, and the error bars are omitted for clarity being of the same order of magnitude of the dot dimensions. On the right side is also reported the anisotropy parameter **β** referred to related panels (see text). For this two-body fragmentation channel the angular distributions of ion products appear to be almost isotropic in the whole investigated photon energy range (all **β** values are ~ 0.0).

**Figure 4:**
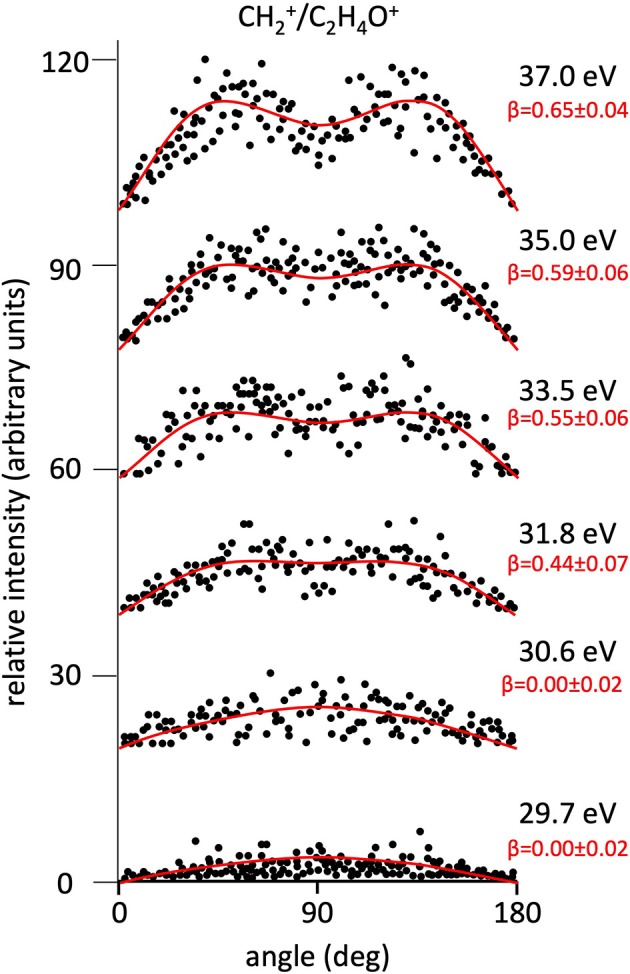
Angular distributions of ${\text{CH}}_{2}^{+}$/C_2_H_4_O^+^ ion pair products formed by Reaction (3) as a function of the photon energy. Dots intensity in the ordinate axis are in arbitrary units, and the error bars are omitted for clarity being of the same order of magnitude of the dot dimensions. On the right side is also reported the anisotropy parameter **β** referred to related panels (see text). For this two-body fragmentation channel the angular distributions of ion products appear to be almost isotropic up to a photon energy of 30.6 eV, whereas at higher photon energy an anisotropic component (**β** > 0.0), which increases as the photon energy increases, must be included in order to obtain a best fit simulation of the experimental data (see text).

**Figure 5:**
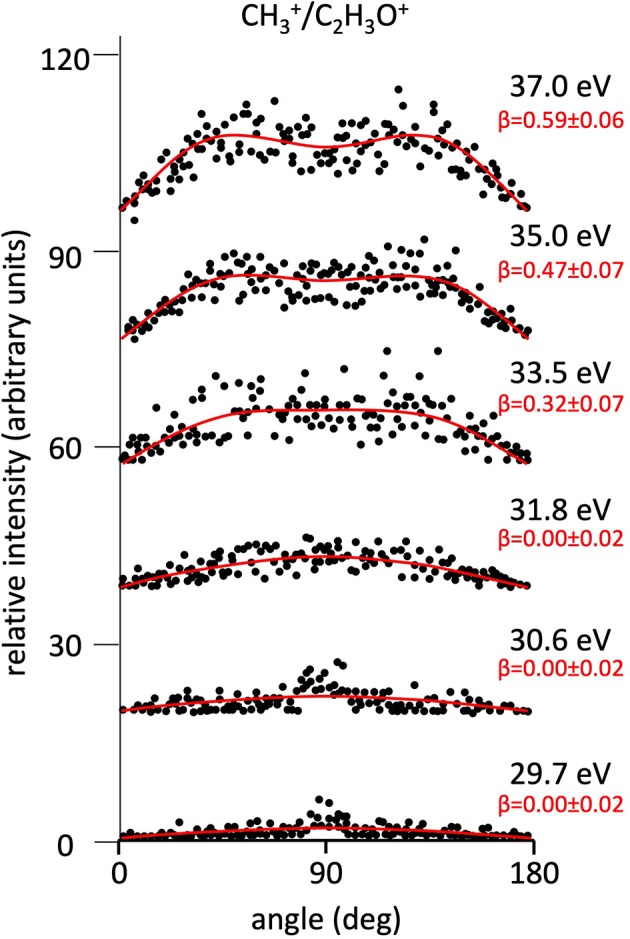
Angular distributions of ${\text{CH}}_{3}^{+}$/C_2_H_3_O^+^ ion pair products formed by Reaction (4) as a function of the photon energy. Dots intensity in the ordinate axis are in arbitrary units, and the error bars are omitted for clarity being of the same order of magnitude of the dot dimensions. On the right side is also reported the anisotropy parameter **β** referred to related panels (see text). For this two-body fragmentation channel the angular distributions of ion products appear to be almost isotropic up to a photon energy of 33.5 eV, whereas at higher photon energy an anisotropic component (**β** > 0.0), which increases as the photon energy increases, must be included in order to obtain a best fit simulation of the experimental data (see text).

**Figure 6:**
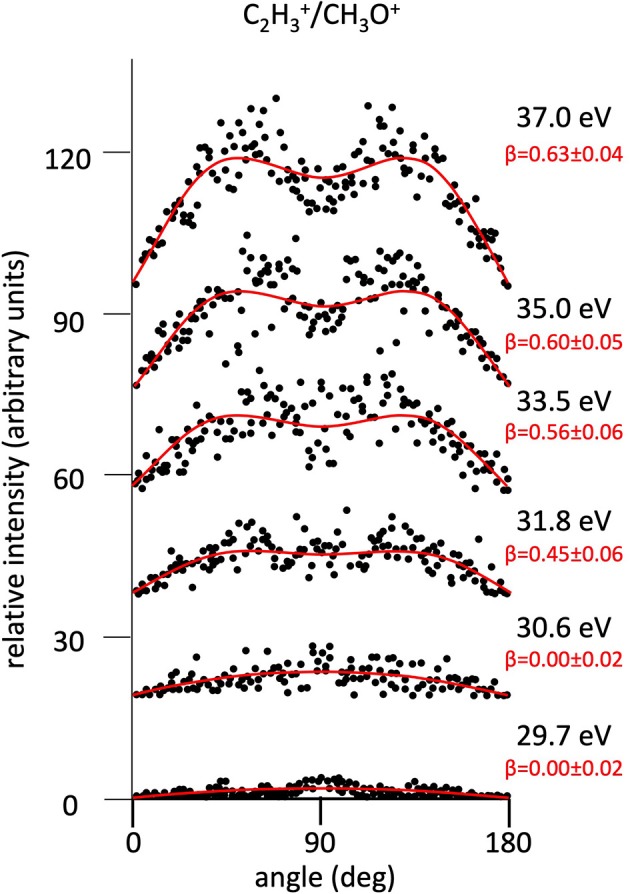
Angular distributions of C_2_${\text{H}}_{3}^{+}$/CH_3_O^+^ ion pair products formed by Reaction (6) as a function of the photon energy. Dots intensity in the ordinate axis are in arbitrary units, and the error bars are omitted for clarity being of the same order of magnitude of the dot dimensions. On the right side is also reported the anisotropy parameter **β** referred to related panels (see text). For this two-body fragmentation channel the angular distributions of ion products appear to be almost isotropic up to a photon energy of 30.6 eV, whereas at higher photon energy an anisotropic component (**β** > 0.0), which increases as the photon energy increases, must be included in order to obtain a best fit simulation of the experimental data (see text).

**Figure 7:**
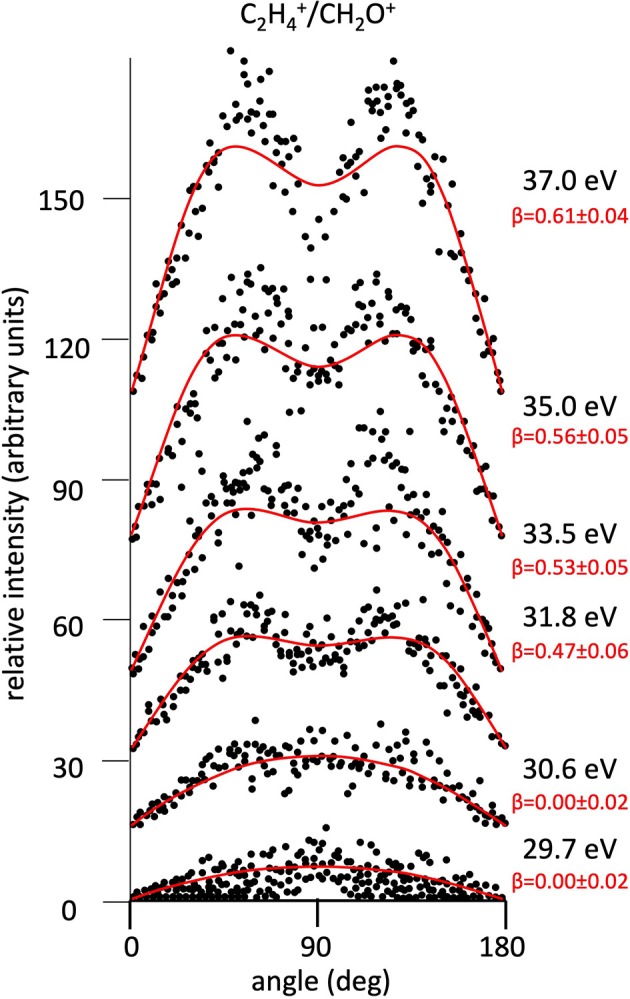
Angular distributions of C_2_${\text{H}}_{4}^{+}$/CH_2_O^+^ ion pair products formed by Reaction (2) as a function of the photon energy. Dots intensity in the ordinate axis are in arbitrary units, and the error bars are omitted for clarity being of the same order of magnitude of the dot dimensions. On the right side is also reported the anisotropy parameter **β** referred to related panels (see text). For this two-body fragmentation channel the angular distributions of ion products appear to be almost isotropic up to a photon energy of 30.6 eV, whereas at higher photon energy an anisotropic component (**β** > 0.0), which increases as the photon energy increases, must be included in order to obtain a best fit simulation of the experimental data (see text).

The important physical meaning of such an equation concerns the evaluation of the **β** term whose determination is crucial to obtain a complete description of the microscopic stereodynamics of fragmentation processes in double photoionization experiments involving linearly polarized light, as the one presented here. In fact, in Equation (8), the differential and total cross sections of the two-body fragmentation process under study are ***I(*θ*)*** and **σ_*tot*_**, respectively; **θ** represents the angle between the light polarization vector direction and the velocity vector of the recorded fragment ion. In general, the anisotropy parameter can assume values in the −1 ≤ **β** ≤ 2 range, depending on the recorded distribution of the final fragment ions. In particular, **β** = 0 characterizes an isotropic distribution of ions, while when **β** ranges from −1 up to 2 this means that the emission of product ions, generated by Coulomb explosion of the intermediate molecular dication, changes gradually from a direction perpendicular to the polarization vector of the light (**β** = −1), to a parallel direction (**β** = 2). Figures 2–7 show the angular intensity distributions [which is the I(θ) sin(θ) product] of final ions of all detected dissociative channels with the exception of Reaction (7) which is too weak to allow a reliable data analysis. The curves in red color are the result of a best fit simulation procedure on the experimental data based on Equations (1) and (8) satisfying the conservation of the total angular momentum of two fragment ions. The error in the measurement of the **β** anisotropy parameters and related angular distributions of Figures 2–7 is of about 0.04–0.06 units of **β** being of the same order of magnitude of the dots dimensions, respectively. In order to control the reliability of our experiment, we started with the measurement of the angular distribution for Reactions (2) and (4) that were already determined in a previous experimental attempt (Falcinelli et al., 2019b) obtaining β parameters in very good agreement with the previous ones. After this preliminary check, we recorded new data of angular distribution of final ion pairs for all two-body dissociation Reactions (2)–(7) (see Figures 2–7).

Figure 2 shows the angular distributions for product ions of Reactions (2)–(6) at a fixed photon energy of 37.0 eV with the determination of the relative **β** values by a fitting simulation procedure based on Equations (1) and (8), as discussed above. It clearly appears that at this photon energy all the recorded angular distributions are characterized by a slight anisotropic behavior with a preferential direction of the product ions emission (from the Coulomb explosion of the intermediate (C_3_H_6_O)^2+^ molecular dication) which is characterized by a parallel component respect to the polarization vector of the used synchrotron radiation. This is confirmed by the obtained **β** values ranging between 0.59 and 0.65. The only exception is given by the Reaction (5) whose angular distribution appears to be substantially isotropic with a **β** value almost zero (**β** = 0.08 ± 0.06). On the other hand, Figures 3–7 report the angular distributions for ion pair products of Reactions (2)–(6) as a function of the photon energy at 29.7, 30.6, 31.8, 33.5, 35.0, and 37.0 eV. In the case of the Reaction (5), the Figure 3 clearly shows an isotropic distribution of product ions in the whole investigated photon energy range, with a recorded anisotropy parameter **β** which is almost zero for each analyzed angular distribution. Conversely, the other two-body fragmentation channels [see Reactions (2)–(4), and (6)] appear to be characterized by two different contributions being active in their respective angular distributions of ion pair products (see Figures 4–7): an isotropic contribution which is dominant at lower photon energies (near the threshold energy of the respective channel) with **β** ≈ 0.00, and one anisotropy component (with ions emission preferentially in a parallel direction respect to the polarization vector of the light) increasing as the photon energy increases (with **β** reaching the values ranging between 0.59 and 0.65 at 37.0 eV, the highest investigated photon energy). This observation is an indication that for Reactions (2)–(4), and (6) the intermediate molecular dication of propylene oxide (C_3_H_6_O)^2+^ at low photon energies, near the threshold (on average up to about 31.0 eV), should be formed in its ground electronic state characterized by a lifetime longer than its typical rotational period of the order of 10^−10^-10^−12^ s (Dantus et al., 1990) with a consequent isotropic fragment ions formation by a slow Coulomb explosion (**β** ≈ 0.00 in the related recorded angular distributions of Figures 4–7). As we have already discussed in previous papers (see for example: Alagia et al., 2007), when an anisotropy appears on the angular distribution of ionic fragments recorded in our PEPIPICO experiments, it is the consequence of the fulfillment of two main conditions: (i) the photon absorption must occur when the molecular orbitals involved in the double photoionization have the most favorable alignment respect to the vector's direction of linearly polarized light; (ii) the separation of two ionic fragments following the dissociative double photoionization should take place in time shorter than the rotational period of the molecular dication which is involved in the Coulomb explosion.

Conversely, at higher photon energy values (in general for hν ≥ 31.8 eV) it should be possible the formation of excited vibronic states of the (C_3_H_6_O)^2+^ dication having shorter lifetimes determining the opening and progressive increase of an anisotropic component in the recorded angular distributions (with a respective gradual increase of the anisotropy parameter toward positive values up to roughly about 0.7). Since our experiment probes a time window from about 50 ns up to 2.5 μs, depending on the used experimental set up (Alagia et al., 2012a), in the absence of evidences in our recorded coincidence plots for any traces due to metastable species (Alagia et al., 2012a,b; Falcinelli et al., 2018a), we can only fix an upper limit of ~50 ns for the lifetime of the intermediate propylene oxide (C_3_H_6_O)^2+^ molecular dication formed in our experimental conditions. In the case of our experiment, observed anisotropies represent also a probe that the main fragmentations occur before the randomization of molecular dication direction due to its rotational temperature. According to suggestions by Felker and Zewail (1987), for the present molecular systems the time scale of such randomizations, evaluated considering the rotational temperature and the average rotational molecular constant, assumes the maximum value of few tens of ps. However, our argumentation is only speculative, and, unfortunately, we cannot do something better at this stage of the data analysis. Specific theoretical calculations able to determine structure, energy and symmetry of dication states as well as the electronic state of fragmentation ion products, energy barriers with their dependence on the geometry of the intermediate state should be very important.

**Figure 8:**
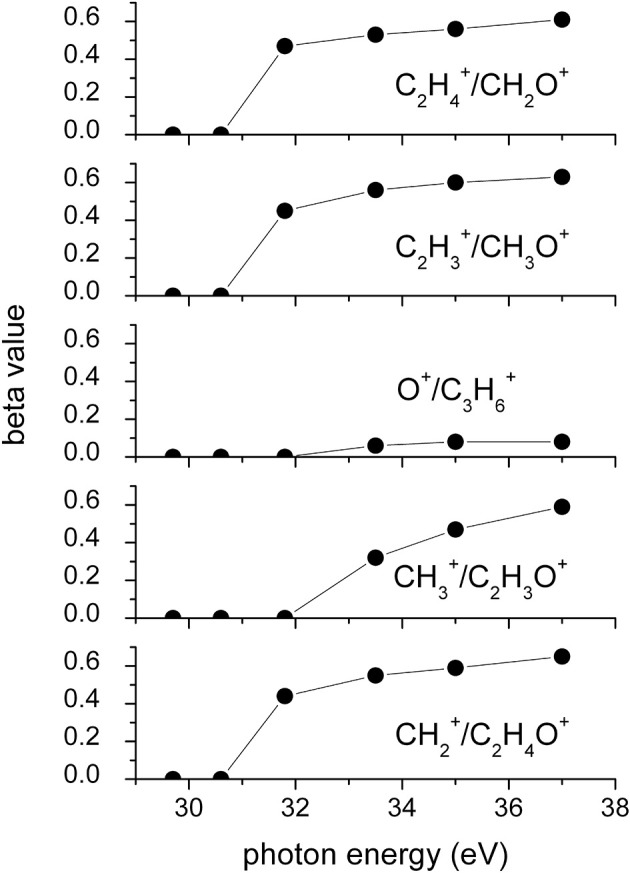
Values of the anisotropy β parameter as obtained by the best fit of the angular distributions recorded for the two-body dissociation Reactions (2)–(6) as a function of the investigated photon energy (see text).

**Figure 9:**
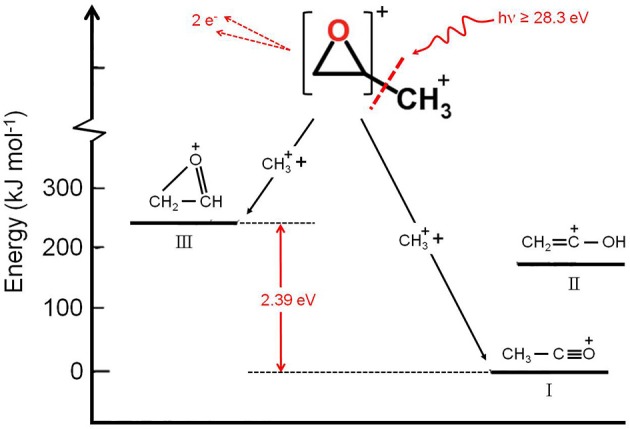
Semiquantitative energetic schematic diagram showing the two alternative microscopic mechanisms for the unimolecular fragmentation of the intermediate propylene oxide (C_3_H_6_O)^2+^ dication forming the two more probable C_2_H_3_O^+^ isomers (see Reaction (4) in the text): the oxiranyl cation (structure III) and the acetyl cation (structure I). In the figure is reported also the third possible stable C_2_H_3_O^+^ isomer, the hydroxyvinyl cation (structure II) whose energetic level is ~1.7 eV above the most stable acetyl cation, and for this reason appears less probable to be formed (see text).

It has to be noted that in such a general behavior [within which we can include all the investigated processes except for Reaction (7)] the Reaction (4) producing ${\text{CH}}_{3}^{+}$ + C_2_H_3_O^+^ shows a different trend. In fact, looking at the Figure 5 it can be seen that the angular distribution of ion products preserves a clear isotropic character with a **β** ≈ 0.00 up to the recorded distribution at a photon energy of 33.5 eV, which is a value quite larger (of about 2.5–3.0 eV) than all the other analyzed cases. Figure 8 reports the anisotropy β parameter obtained from our data analysis (see Figures 2–7) as a function of the investigated photon energy range for all recorded two-body dissociation channels [see Equations (2)–(6)] with the only exception of Reaction (7) since, as mentioned above, the collected signal was too low not allowing a meaningful analysis of the data. From the figure it is evident that the Reaction (4), the one characterized by a bimodal trend in the KER distributions (see Figure 1C), is also the unique two-body dissociation process for which the β parameter starts an anisotropic behavior at a photon energy higher than ~32.0 eV. All the other recorder fragmentation channels, with the exception of Reaction (5) for which **β** is almost zero at all investigated photon energies, show a different trend with an anisotropic behavior that appears for lower photon energy values (hν ≥ 30.6 eV). This particular behavior could be a confirmation of the possibility that two different microscopic mechanisms are operative for such a two-body fragmentation process as we have discussed in a previous paper (Falcinelli et al., 2018a) where a bimodal behavior was found in the total KER distributions recorded for ${\text{CH}}_{3}^{+}$ + C_2_H_3_O^+^ ions produced in Reaction (4) (Falcinelli et al., 2018a,b, 2019b). A portion of such distributions is reported in Figure 1C for two different photon energy values (35.0 and 37.0 eV) and clearly show a bimodality being the experimental data best fitted by a sum of two Gaussian functions whose peak maxima are separated by ≈ 2.1 eV. It is interesting to note that previous ab initio molecular orbital calculations allowed Nobes et al. (1983) to determine structures and stabilities of various C_2_H_3_O^+^ isomers. These authors found that only three different isomers of C_2_H_3_O^+^ can be stable and observable species in gas-phase experiments. They are shown in Figure 9. In order of their decreasing energetic stability, the most stable is the acetyl cation [CH_3_−C = O]^+^ (structure I in Figure 9) which is followed by the hydroxyvinyl cation [CH_2_ = C−OH]^+^ (structure II in Figure 9), and finally the less stable is the oxiranyl cation [CH_2_−CH−O]^+^ (see structure III in Figure 9). The first two isomers are characterized by a linear structure, while the oxiranyl is a cyclic isomer with a triangular [C···O···C] ring (Nobes et al., 1983), as it is shown in Figure 9. Immediately after the publication of these calculations, experiments were performed by Burgers et al. demonstrating the unequivocal identification of all three mentioned C_2_H_3_O^+^ isomers in gas-phase (Burgers et al., 1983). It has to be noted that the difference in the energetic stability between the most stable acetyl cation and the two other isomers is not very different. The acetyl cation is ≈ 1.87 eV more stable respect to the hydroxyvinyl cation, while the oxiranyl cation is located at ≈ 2.53 eV above the acetyl one (see Figure 9) (Nobes et al., 1983). Both pairs of energy values are very close to the difference between peak maxima of the two Gaussian functions used to best fit our recorded KER (see Figure 1C). In particular, this energetic value of about 2.1 ± 0.3 eV is the difference in the translational energy of the two possible C_2_H_3_O^+^ ions produced via two different microscopic mechanisms, following the Coulomb explosion of the intermediate (C_3_H_6_O)^2+^ dication. It seems to fit better with the formation of: (i) the linear acetyl cation with a higher translational energy content (to which are associated the KER distributions centered at about 6.1 eV in Figure 1C), since it is the more stable isomer one; (ii) the cyclic oxiranyl isomer (to which are related the less intense KER distributions of Figure 1C located at about 4.0 eV) which is produced with a lower kinetic energy respect to the previous one, being ~2.53 eV less stable than the other. Obviously, we cannot exclude the possible formation of hydroxyvinyl cation, even if the two following considerations make this hypothesis less probable: (i) its production from the Coulomb explosion of the (C_3_H_6_O)^2+^ dication should involve a hydrogen shift toward the O atom of the propylene oxide ring resulting in its breakage and openness; (ii) the energetic difference of ~1.87 eV in the stability between acetyl and hydroxyvinyl isomers as calculated by Nobes et al. (1983) do not fit properly with the difference in the translational energy characterizing the formation of C_2_H_3_O^+^ ions coming out by the two different recorded mechanisms: the bimodality in the total KER distributions displays that the difference in the translational energy of the two formed C_2_H_3_O^+^ isomers (one of which is always the more stable acetyl cation) could reach an upper limit of about 4.1–4.2 eV which is reasonable in the case of the oxiranyl cation formation, but it appears too high for the possible formation of the hydroxyvinyl isomer. It has to be noted that in the microscopic fragmentation dynamics of the intermediate (C_3_H_6_O)^2+^ dication a H-migration could be possible prior to dissociation. In order to clarify this point, the estimation of the H-migration rate toward the formation of the two different C_2_H_3_O^+^ final isomers, and the assessment of the role of electronically excited states of (C_3_H_6_O)^2+^ dication should be very important. To this aim further experimental (for example, using labeled isotopic variants of propylene oxide) and accurate theoretical efforts are mandatory.

In order to clarify this qualitative speculation it should be highly helpful to perform further theoretical calculations on the structure and energetic stability of the intermediate propylene oxide dication, as well as of all possible ionic species that can be formed following its unimolecular two-body fragmentation by Coulomb explosion. Similar calculations have been carried out in our laboratory for simpler systems (Teixidor et al., 2003; Candori et al., 2007; Leonori et al., 2009a,b; Rosi et al., 2012; Skouteris et al., 2015).

## Conclusions

This study is important as no data on the propylene oxide (C_3_H_6_O)^2+^ molecular dication energetics and on its nuclear dissociation dynamics are available. This information is required for further experimental and theoretical investigations of the interaction between chiral molecules and linearly or circularly polarized radiation in future projects. In order to investigate in detail stereodynamical mechanisms for the discrimination of enantiomers, a future extension of our experiments will aim at identifying possible differences in the angular and energy distribution of fragment ions and ejected photoelectrons, observable at different photon energies, coming out from the interaction of circularly polarized synchrotron light with propylene oxide and other simple chiral molecules, such as the 2-butanol, available as enantiomerically pure. The present paper reports on the measure of angular distributions of ion products as a function of the photon energy for each investigated two-body fragmentation channels produced in a double photoionization experiment involving propylene oxide.

All recorded angular distributions are characterized by a slight anisotropic behavior with a preferential direction of the product ions emission which is characterized by a parallel component respect to the polarization vector of the used synchrotron radiation. This is confirmed by the obtained **β** values ranging between 0.59 and 0.65. The only exception is given by the Reaction (5) whose angular distribution appears to be substantially isotropic with a **β** value almost zero (**β** = 0.08 ± 0.06).

The data analysis allowed the determination of the anisotropy parameter **β** for each investigated dissociation reaction as well as its behavior as the photon energy changes over the 18.0–37.0 investigated range. This seems to be consistent with the hypothesis of the possibility of two different microscopic mechanisms for the fragmentation channel producing ${\text{CH}}_{3}^{+}$/C_2_H_3_O^+^ ion pairs, already observed in their KER distributions in recent experiments (Falcinelli et al., 2018a,b). On the basis of previous theoretical calculations (Nobes et al., 1983) and experimental evidences (Burgers et al., 1983), we can argue that following the double photoionization of propylene oxide, different electronic states of the intermediate (C_3_H_6_O)^2+^ dication could be formed with their subsequent two-body fragmentation toward the final formation of different stable isomers of C_2_H_3_O^+^. From energetic considerations on our recorded data, the more probable microscopic mechanism seem to be those that lead to the formation of the acetyl and oxiranyl pair of isomers. Moreover, we cannot exclude the formation of the third stable possible C_2_H_3_O^+^ isomer, the hydroxyvinyl cation, even if it appears less probable. To understand comprehensively the microscopic dynamics of fragmentation of doubly photoionized propylene oxide, three are the possible strategies to follow: (i) to perform further experiments using isotopically labeled precursor molecules; (ii) to carry out theoretical calculations on structure and energetic stability of either propylene oxide (C_3_H_6_O)^2+^ dication and final ions coming out from its Coulomb explosion; (iii) to adopt a new original methodology developed by our research group in order to fully describe the stereo-dynamics of dissociative double ionization reactions as recently applied to depict the reactivity of auto-ionization processes at a state to state level (Falcinelli et al., 2018c, 2019a). Our research group will be involved in both these directions during next future.

## Author Contributions

SF, MA, and LS analyzed the results. All authors planned and managed the experiment, made discussion about the results and participated in writing the manuscript.

### Conflict of Interest Statement

The authors declare that the research was conducted in the absence of any commercial or financial relationships that could be construed as a potential conflict of interest.
